# Assessment of EN-RAGE, sRAGE and EN-RAGE/sRAGE as potential biomarkers in patients with autoimmune hepatitis

**DOI:** 10.1186/s12967-020-02556-w

**Published:** 2020-10-09

**Authors:** Rui Wu, Yan Liu, Ruyu Yan, Xiaoyu Liu, Liang Duan

**Affiliations:** 1 Department of Laboratory Medicine, The First Affiliated Hospital of Chongqing Medical University, No.1 You Yi Road, Yuan Jia Gang, Yu Zhong District, Chongqing, China; 2 Key Laboratory of Diagnostic Medicine Designated by the Chinese Ministry of Education, Chongqing Medical University, Chongqing, China; 3 Department of Rheumatology and Immunology, The Second Affiliated Hospital of Chongqing Medical University, Chongqing, China; 4 School of Biomedical Sciences, Faculty of Medicine, The Chinese University of Hong Kong, Shatin, N.T., Hong Kong, China; 5 Department of Laboratory Medicine, The Second Affiliated Hospital of Chongqing Medical University, No.74 linjiang Road, Yu Zhong District, Chongqing, China

**Keywords:** EN-RAGE, sRAGE, Autoimmune hepatitis, Inflammatory-immune response

## Abstract

**Background:**

Autoimmune hepatitis (AIH) is a liver disease characterized by the autoimmune-induced injury of hepatocytes which can lead to cirrhosis and hepatic failure. The diagnosis and disease management of AIH patients remain challenging due to the diversity of clinical phenotypes and the presence of confounders such as alcohol and viruses. Recently, EN-RAGE and sRAGEs have been implicated in inflammatory-immune response. Nonetheless, their natural behaviour and relationship to disease activity as well as clinical predictive values in AIH development or therapy-induced remission have not been reported.

**Methods:**

Sixty-seven AIH patients and thirty gender- and age-matched healthy controls (HC) were enrolled. The serum concentrations of EN-RAGE, sRAGE and their ratio (EN-RAGE/sRAGE) in these subjects were measured by ELISA. Besides, the correlations of three parameters with clinical features and therapeutic response were analyzed, respectively. Furthermore, their potential predictive values for monitoring the AIH progression and therapeutic response were also evaluated.

**Results:**

Higher serum EN-RAGE, lower sRAGE and higher EN-RAGE/sRAGE value were observed in AIH patients. EN-RAGE and sRAGE as well as EN-RAGE/sRAGE were correlated with liver necroinflammation parameters, cirrhosis occurrence and therapeutic response. In addition, we identified that EN-RAGE/sRAGE, EN-RAGE and sRAGE had valuable predicting power for AIH patients, AIH patients with normal ALT and cirrhosis incidence, respectively. More importantly, EN-RAGE/sRAGE also exerted predicting power for the remission in AIH patients.

**Conclusions:**

AIH patients rendered distinct patterns of serum EN-RAGE, sRAGE or EN-RAGE/sRAGE compared to healthy controls. Moreover, these three parameters exhibited potentials as novel biomarkers for AIH diagnosis and prognosis evaluation.

## Background

Autoimmune hepatitis (AIH) is a liver-specific autoimmune disease characterized by hypergammaglobulinemia, increased circulating autoantibodies, transaminases, and histological evidence of interface hepatitis [1]. AIH occurs in all ethnicities and ages, with a female predominance. Intolerance to self-antigens followed by destruction of hepatic parenchyma by T lymphocyte-mediated response is identified as the key pathogenic mechanisms [2]. Untreated AIH can lead to liver cirrhosis and terminal failure [3]. So far, autoantibodies against nuclear antigens (ANA) or smooth muscle antibodies (ASMA), combination of liver kidney type 1 (LKM1) and other liver biochemical indexes such as alanine aminotransferase (ALT), aspartate aminotransferase (AST), gamma globulin and immunoglobulin G (IgG) are widely used as biomarkers for AIH diagnosis. However, the sensitivity and specificity of these markers for AIH diagnosis and evaluation of disease severity or therapeutic effect are still very limited, leading to misdiagnosis, inaccurate disease assessment and missing the treatable stage of AIH [4]. Therefore, unveiling new quantifiable biomarkers that are closely related to its pathogenesis as well as accurately reflects disease severity and therapeutic response is urgently needed.

The receptor for advanced glycation end products (RAGE), a receptor expressed on the cell surface, can be bound by multiple types of damage-associated molecular patterns (DAMPs), including S100/calgranulins, high-mobility group box 1 (HMGB1) and advanced glycation end products (AGEs), leading to the activation of the downstream cascades which is responsible for inflammatory-immune response [5]. Extracellular newly identified receptor for advanced glycation end products binding protein (EN-RAGE), also known as S100A12, is a calcium-binding proinflammatory protein predominantly secreted by activated granulocytes, macrophages and lymphocytes [6]. When secreted extracellularly, EN-RAGE, as one type of the DAMPs, induces persistent inflammatory-immune response by binding to RAGE followed by activation of intracellular signal cascades in lymphocytes and production of proinflammatory cytokines [7]. Increased serum EN-RAGE levels have been detected in multiple inflammatory-immune disorders [8, 9]. However, it is still unclear how EN-RAGE participates in the inflammatory-immune process and whether it can serve as a useful indicator for AIH progression.

Soluble RAGE (sRAGE), a splicing variant of the full-length receptor, is a soluble isoform containing only the RAGE extracellular domain formed by proteolytic cleavage. sRAGE counteracts DAMPs-RAGE interaction by sequestering and eliminating DAMPs [10]. Therefore, sRAGE can function as a “decoy” by binding to EN-RAGE and prevent the ligation between EN-RAGE and RAGE, which attenuate the inflammatory-immune response. Clinically, the serum levels of sRAGE are reduced in several types of auto-immune diseases, such as systemic lupus erythematosus [11], hashimoto's thyroiditis [12] and guillain-barré syndrome [13]. AIH is also an autoimmune disease with hepatocellular injury caused by liver-infiltrated T cells including T helper type 1 (Th1), Th2 and Th17 cells [1, 14]. T lymphocyte activation and differentiation was reported to be regulated by RAGE ligation by an in vivo mouse study [15]. Recent studies showed a protective role of sRAGE during the process of hepatocellular injury [16, 17]. However, the serum level of sRAGE in AIH and its underlying relationship with disease activity remain elusive.

The activation of RAGE by EN-RAGE and the antagonism by sRAGE results in inflammatory-immune responses augmentation and mitigation, respectively. Nonetheless, to date, the relationship of serum EN-RAGE or sRAGE levels with disease activity has not been well defined in AIH. In this study, we determined the serum levels of EN-RAGE and sRAGE as well as their ratio (EN-RAGE/sRAGE) and explored their correlation with clinical parameters in a well-defined cohort of AIH patients, aiming to explore whether EN-RAGE, sRAGE or EN-RAGE/sRAGE can be used as potential disease biomarkers during AIH progression.

## Methods

### Patients

Sixty-seven newly diagnosed AIH patients were prospectively recruited from the First and Second Affiliated Hospital of Chongqing Medical University from May 2016 to May 2018. Patients were not included if they were detected with other concomitant disease entities, including virus hepatitis, drug-induced hepatic injury, genetic and metabolic liver disease, hepatic malignancies, primary biliary cholangitis (PBC), primary sclerosing cholangitis (PSC) and other autoimmune diseases. AIH was diagnosed according to the simplified criteria suggested by the IAIHG [18]. Clinical characteristics of patients were recorded at the time of AIH diagnosis. The patients were treated with decreasing doses of azathioprine and prednisone. Remission was defined according to the international guidelines. Biochemical remission was defined as the normalization of alanine aminotransferase (ALT), aspartate aminstransferase (AST) and IgG levels after treatment. Additionally, thirty age and gender-matched healthy controls [Age median and interquartile range (IQR): 48 (18); female, n (%): 49 (73.2%)] who did not have evidence of liver diseases or other chronic disorders were enrolled as healthy controls (HCs). All serum samples from thepatients and HCs were stored at − 80° until use. The study was approved by the Institutional Ethics Committee for human studies at Chongqing Medical University, Chongqing, China. All the procedures were following the Declaration of Helsinki. Patient characteristics were summarized in Table 1.

**Table 1:** The characteristics of enrolled individuals

Parameters	AIH (n = 67)	HC (n = 30)
Sex as female/male, n (%)	49 (73.20)/18 (26.80)	22 (73.33)/8(26.66)
Age (years)	50 (20)	48 (18)
Laboratory findings		
ALT (U/L)	112 (103)	NA
AST (U/L)	64 (47)	NA
TB (µmol/L)	10.7 (10.1)	NA
ALB (g/L)	35.8 (13)	NA
IgG (g/L)	24.43 (12.78)	NA
Autoimmune serology, n (%)		
ANA	16 (23.88)	NA
SMA	18 (26.86)	NA
ANA + SMA	12 (17.91)	NA
Cirhosis, n (%)		
Yes	39 (58.20)	NA
No	28 (41.80)	NA

For age, Age, ALT, AST and TB, data are presented as median (IQR). *ANA* anti-nuclear antibody; *ASMA* anti-smooth muscle antibody; *ALB* albumin; *TB* total bilirubin; *NA* not applicable

### Autoimmune serology and hepatic biochemical indexs and assessment

Antinuclear antibodies such as ANA and ASMA were measured by an automatic indirect immunofluorescence device Sprinter XL (EUROIMMUN, Cermany) and analyzed by automatic EUROP attern Microscope (EUROIMMUN, Cermany). Hepatic biochemical indexs such as alanine aminotransferase (ALT), aspartate aminstransferase (AST), albumin (ALB) and total bilirubin (TB) were measured by HITACHI 7600 (HITACHI, Japan). Immunoglobulin G (IgG) levels were detected by cobas c311 analyzer (Roche, Switzerland).

### Enzyme-linked immunosorbent assay

Serum samples were analyzed by commercial human EN-RAGE (Cusabio, China) and sRAGE enzyme-linked immunosorbent assay kits (JYM, China) used according to the manufacturer’s instructions. Samples were run in duplicate.

### Statistical analysis

Data were analyzed using SPSS 17.0. Mann–Whitney or Kruskal–Wallis test was performed to determine significance of EN-RAGE and sRAGE as well as their ratio (EN-RAGE/sRAGE) in AIH patients with various clinical and biochemical features. Correlation coefficients (r) were calculated using spearman correlation. Paired t test was used for EN-RAGE and sRAGE as well as EN-RAGE/sRAGE in AIH with remission analysis. Receiver operating characteristic (ROC) curves were generated to classify patients in different groups, as well as for the evaluation of predicting power for serum EN-RAGE and sRAGE as well as EN-RAGE/sRAGEvia calculation of the area under the ROC curve (AUC), sensitivity and specificity according to standard formulas. All the data represents the median and interquartile range (IQR); A *p* value < 0.05 was considered statistically significant.

## Results

### Serum levels of EN-RAGE, sRAGE and their ratio (EN-RAGE/sRAGE) in AIH patients

**Fig. 1:**
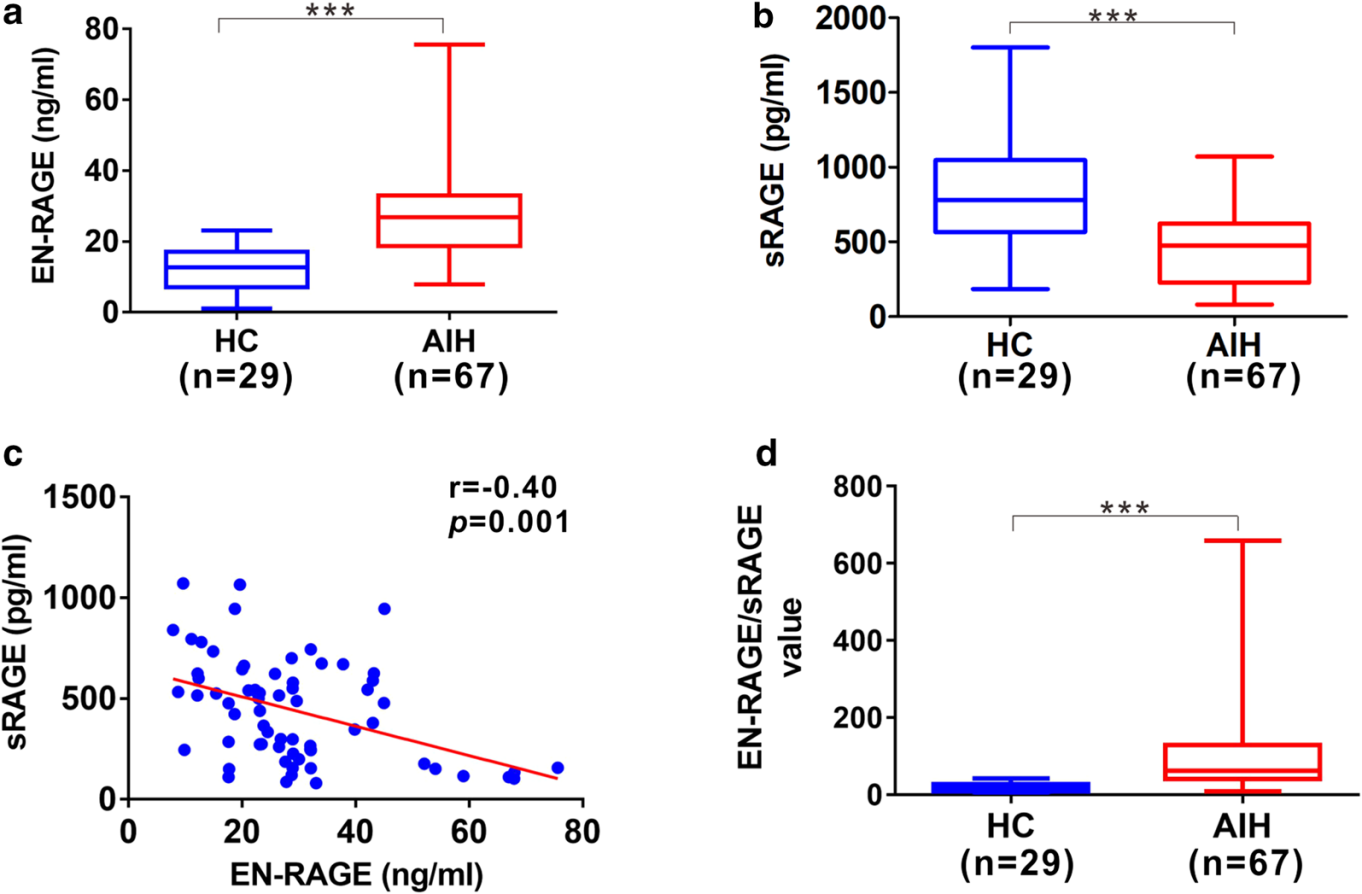
Serum levels of EN-RAGE and sRAGE and their ratio (EN-RAGE/sRAGE) in AIH. **a** ELISA analysis of serum EN-RAGE levels from HC and AIH patients. **b** ELISA analysis of serum sRAGE levels from HC and AIH patients. **c** Correlation between serum EN-RAGE and sRAGE in AIH patients. **d** Analysis of EN-RAGE/sRAGE value in HC and AIH patients. Data represents the median (IQR). *n* number. ***p < 0.001

To assess whether serum levels of EN-RAGE and sRAGE are abnormally altered in AIH, we analyzed their levels in AIH and compare them to healthy controls (HC). The serum level of EN-RAGE was significantly higher in AIH patients [26.8 (14.31) ng/ml] than that of HC group [12.64 (10.06) ng/ml] (Fig. 1a). Contrarily, serum level of sRAGE was remarkably lower in AIH group [476.5 (395.8) pg/ml] than that of HC group [781.3 (482.2) pg/ml] (Fig. 1b). Additionally, serum level of EN-RAGE was negatively correlated with sRAGE (Fig. 1c). We then analyzed their ratio (EN-RAGE/sRAGE) in AIH, which showed that EN-RAGE/sRAGE was prominently higher in AIH group [61.54 (88.38)] compared to HC group [15.07 (18.78)] (Fig. 1d). AIH has a female predilection, we analyzed EN-RAGE, sRAGE and their ratio (EN-RAGE/sRAGE) in AIH patients with different genders. However, all the three parameters showed no significant differences between the male group [26.14 (9.84) ng/ml; 411.7 (380.4) pg/ml; 68.44 (98.36)] and female group [27.8 (21.76) ng/ml; 478.3 (402.8) pg/ml; 61.54 (84.71)] (Additional file 1: Fig.S1).

### Circulating EN-RAGE and sRAGE as well as their ratio (EN-RAGE/sRAGE) in AIH patients with seropositive and seronegative pathogenic autoantibodies

**Fig. 2:**
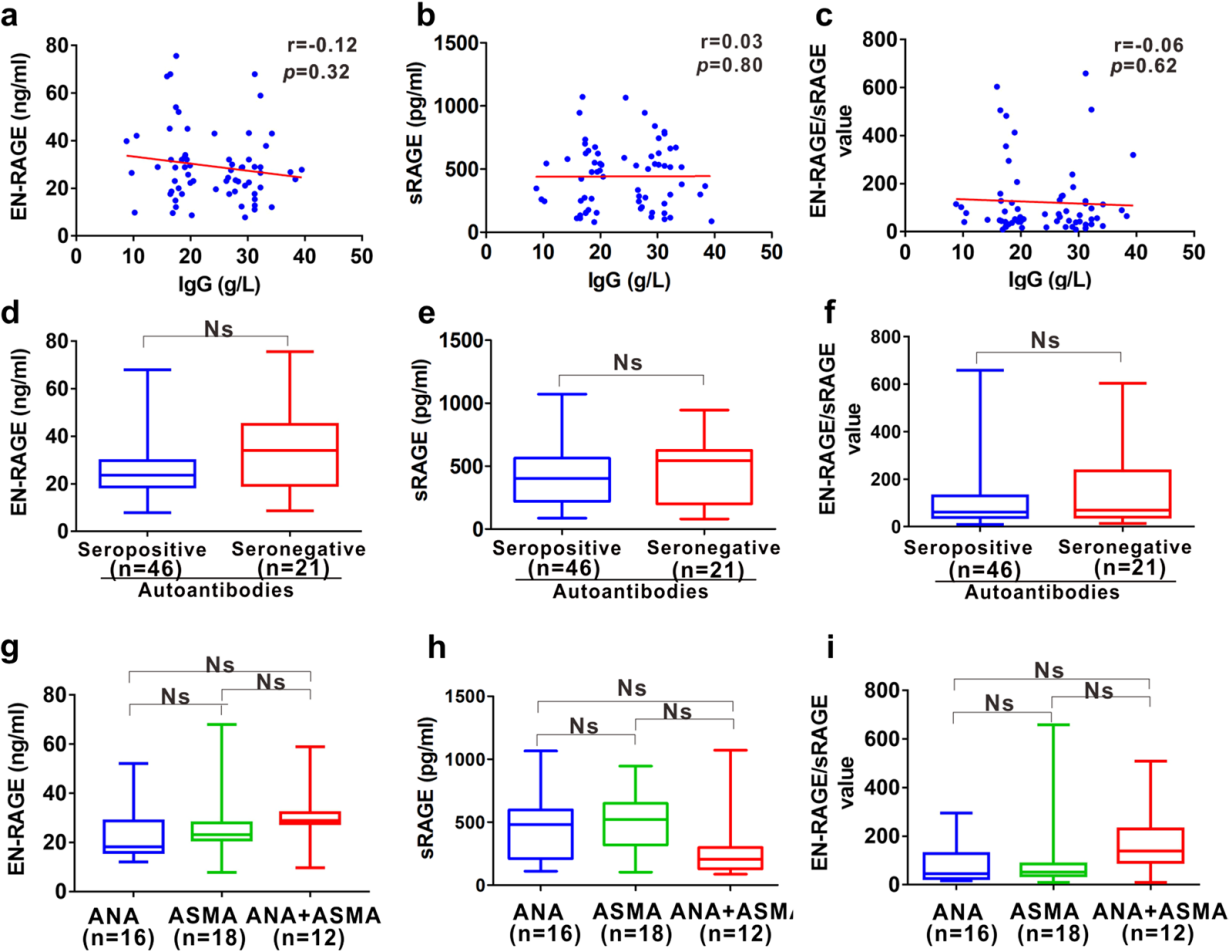
Relationship of EN-RAGE, sRAGE or EN-RAGE/sRAGE with pathogenic autoantibodies in AIH patients.** a**–**c** Correlation between serum EN-RAGE (**a**), sRAGE (**b**) or EN-RAGE/sRAGE (**c**) and IgG levels in AIH patients.** d**–**f** Distribution of serum levels of EN-RAGE (**d**), sRAGE (**e**) and EN-RAGE/sRAGE value (**f**) in AIH patients with seropositive and seronegative pathogenic autoantibodies. **g**–**i** Distribution of serum levels of EN-RAGE (**g**), sRAGE (**h**) and EN-RAGE/sRAGE (**i**) in AIH patients with ANA, ASMA or ANA + ASMA. Data represents the median (IQR). *n* number. *Ns* no statistical significance

AIH is defined by increased serum level of IgG and the presence of autoantibodies, nonetheless, none of the EN-RAGE, sRAGE nor EN-RAGE/sRAGE was correlated with IgG level (Fig. 2a-c). Since the circulating autoantibodies are the hallmarks of AIH diagnosis, the relationships between EN-RAGE, sRAGE or EN-RAGE/sRAGE and the presence of autoantibodies were determined. Whereas, the three parameters showed no statistic significant changes in autoantibodies-seropositive [34 (25.6) ng/ml; 544 (424.2) pg/ml; 69 (254.72)] AIH patients compared to seronegative [23.63 (11.04) ng/ml; 401.4 (342.6) pg/ml; 61.16 (90.71)] AIH patients (Fig. 2d-f). As ANA and ASMA are used as key serum diagnostic antibodies for AIH, we further determined the abundance of EN-RAGE, sRAGE or EN-RAGE/sRAGE in patients with different subtypes of autoantibodies. However, no significant differences were observed among ANA group, ASMA group and ANA + ASMA group (Fig. 2g-i).

### Correlations of EN-RAGE, sRAGE and EN-RAGE/sRAGE with hepatic biochemical indexes

Hepatic biochemical necroinflammation indexes such as alanine aminotransferase (ALT), aspartate aminotransferase (AST), albumin (ALB), total bilirubin (TB) are crucial factors for reflecting liver functional state. We further analyzed the relationship between hepatic biochemical parameters and EN-RAGE, sRAGE or their ratio (EN-RAGE/sRAGE), respectively in AIH patients. Both serum EN-RAGE and the EN-RAGE/sRAGE ratio showed a positive correlation with ALT and AST but not ALB or TB in AIH patients (Table 2). In contrast, serum sRAGE was negatively correlated with ALT and AST but not with ALB and TB in AIH patients (Table 2).

**Table 2:** Correlations of EN-RAGE, sRAGE and their ratio (EN-RAGE/sRAGE) with hepatic biochemical indexs

	ALT (U/L)		AST (U/L)		ALB (g/L)		TB (µmol/L)	
	r	*p* value	r	*p* value	r	*p* value	r	*p* value
EN-RAGE (ng/ml)	0.30	0.01	0.27	0.02	− 0.16	0.20	− 0.16	0.20
sRAGE (pg/ml)	− 0.27	0.02	− 0.30	0.02	− 0.06	0.61	− 0.06	0.61
EN-RAGE/sRAGE	0.32	0.009	0.32	0.009	− 0.06	0.61	− 0.06	0.62

*r* correlation coefficient r. *p* values < 0.05 are considered as significant

### Serum levels of EN-RAGE, sRAGE and EN-RAGE/sRAGE in AIH patients with cirrhosis

**Fig. 3:**
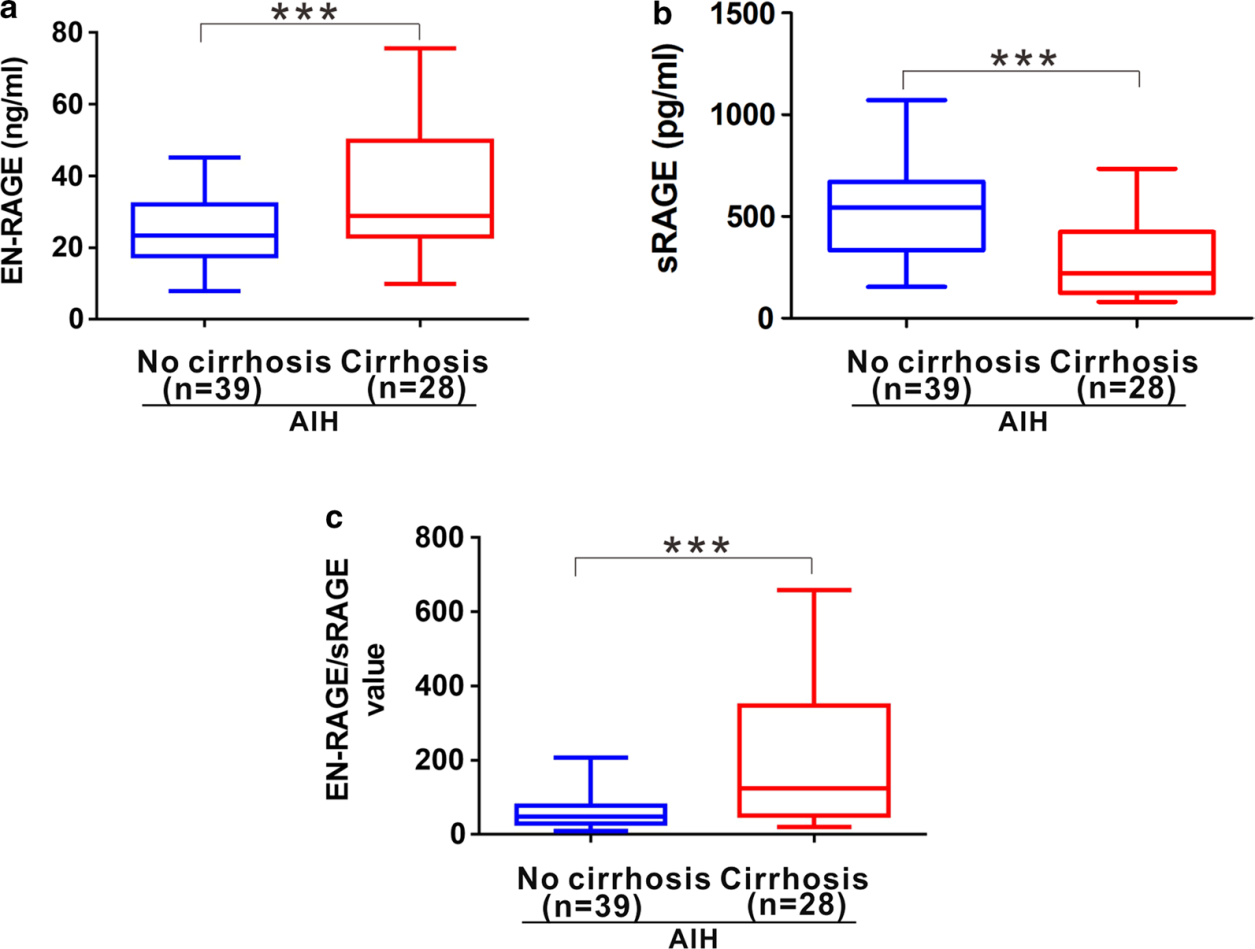
Serum levels of EN-RAGE and sRAGE as well as EN-RAGE/sRAGE in AIH patients with and without cirrhosis. **a** Distribution of serum levels of EN-RAGE in AIH patients with and without cirhosis. **b** Distribution of serum levels of sRAGE in AIH patients with and without cirhosis. **c** Distribution EN-RAGE/sRAGE in AIH patients with and without cirhosis. *n* number. Data represents the median (IQR). ***p < 0.001

Autoimmune hepatitis without proper treatment can develop into liver cirrhosis. Therefore, we detected and analyzed the serum levels of EN-RAGE and sRAGE as well as their EN-RAGE/sRAGE value in AIH patients with and without liver cirrhosis. AIH patients with liver cirrhosis exhibited higher EN-RAGE levels [28.83 (26.73) ng/ml] than AIH patients without cirrhosis [23.46 (14.4) ng/ml] (Fig. 3a). Contrarily, AIH patients with liver cirrhosis showed lower sRAGE levels [221.7 (300.3) pg/ml] than the patients without cirrhosis [543 (336.3) pg/ml] (Fig. 3b). As a result, AIH patients with liver cirrhosis possessed higher EN-RAGE/sRAGE ratio [124.6 (296.15)] than the patients without cirrhosis [47.7 (48.01)] (Fig. 3c).

### Recovery phase of AIH patients possess lower EN-RAGE and EN-RAGE/sRAGE but higher sRAGE

**Fig. 4:**
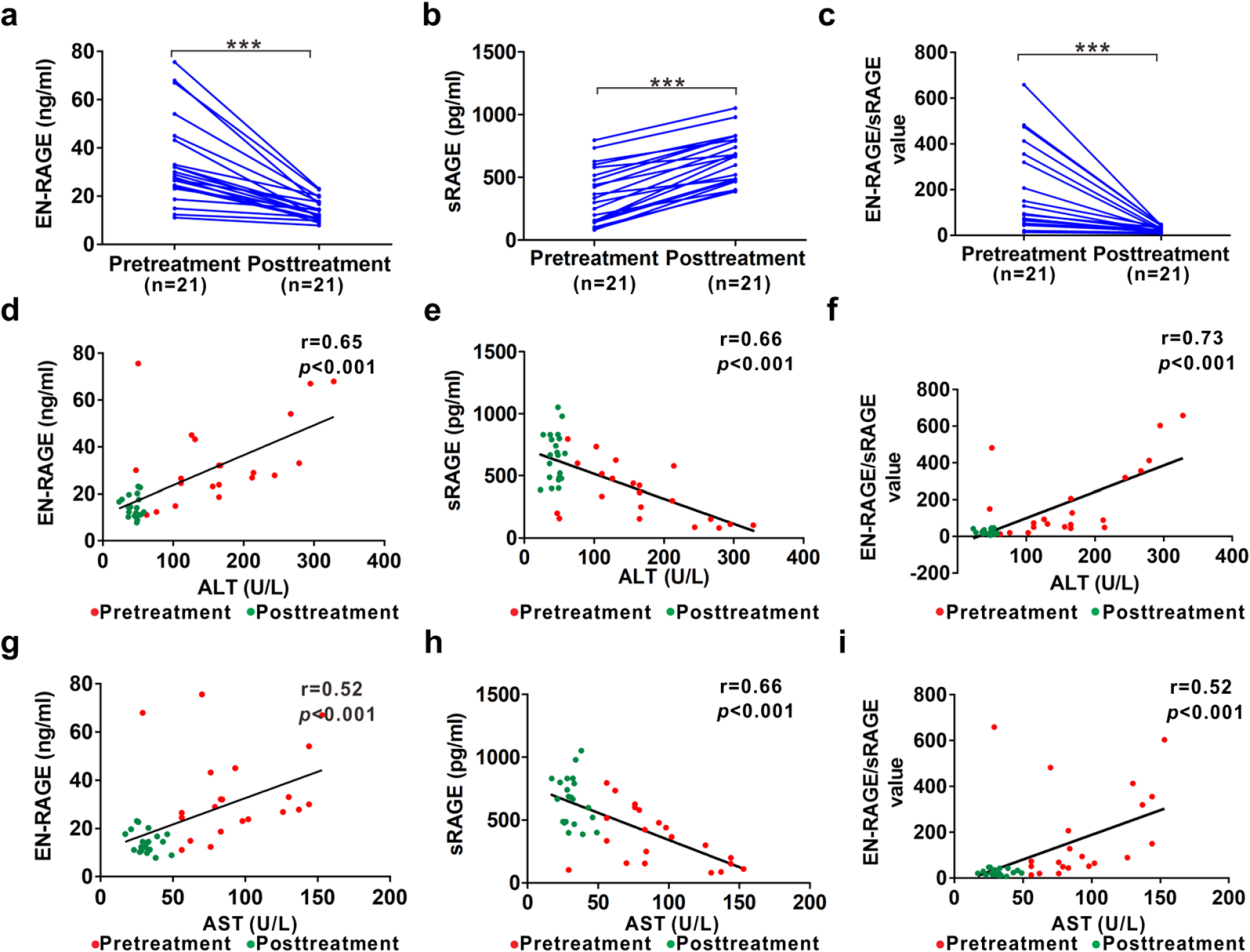
Serum levels of EN-RAGE and sRAGE as well as EN-RAGE/sRAGE before and after therapy of AIH patients. **a** Serum levels of EN-RAGE in AIH patients before and after therapy. **b** Serum levels of sRAGE in AIH patients before and after therapy. **c** EN-RAGE/sRAGE in AIH patients before and after therapy. **d**–**f** Correlation of serum EN-RAGE (**d**), sRAGE (**e**) or EN-RAGE/sRAGE value (**f**) with ALT in AIH patients before and after therapy. **g**–**i** Correlation of serum EN-RAGE (**g**), sRAGE (**h**) or EN-RAGE/sRAGE (**i**) with AST in AIH patients before and after therapy. *n* number. ***p < 0.001

To further investigate the relationship of between therapeutic effect and EN-RAGE, sRAGE or EN-RAGE/sRAGE, twenty-one AIH patients who achieved disease remission were enrolled in this study. By monitoring serum EN-RAGE, sRAGE, EN-RAGE/sRAGE value and clinical parameters for these patients, we found that posttreatment group showed a striking reduction of ALT and AST but improved level of ALB, implying that there is a mitigation of liver damage and recovery of liver functions. Moreover, IgG level was decreased in the posttreatment group, suggesting that there is a disease remission upon the AIH therapy (Table 3). Interestingly, after the therapeutic treatment for AIH, serum levels of EN-RAGE were dramatically diminished while sRAGE were elevated (Fig. 4a, b). As expected, EN-RAGE/sRAGE value was also decreased in the remission phase of AIH patients (Fig. 4c). More importantly, EN-RAGE, sRAGE, EN-RAGE/sRAGE were significantly correlated with ALT and AST during the whole process of therapy, respectively (Fig. 4d-i).

**Table 3:** Effect of treatment on the values of clinical measures in AIH patients

Parameters	Before treatment	After treatment	*p* value
n	21	21	NA
Age (Years)	45 (20)	45 (20)	NA
Gerder (F/M)	16/5	16/5	NA
ALT (U/L)	165 (122)	48 (13)	< 0.001
AST (U/L)	83 (62)	31 (9.5)	< 0.001
ALB (g/L)	34 (11.75)	45.6 (8.4)	< 0.001
TB (µmol/L)	8 (14.55)	12.6 (12.2)	> 0.05
IgG (g/L)	20.49 (18.96)	11.34 (5.94)	< 0.001

For age, gender, ALT, AST, ALB, TB and IgG, data are presented as median (IQR); *NA* not applicable; *p* values < 0.05 are considered as significant

### Diagnostic power of serum EN-RAGE, sRAGE and EN-RAGE/sRAGE for AIH progression

**Fig. 5:**
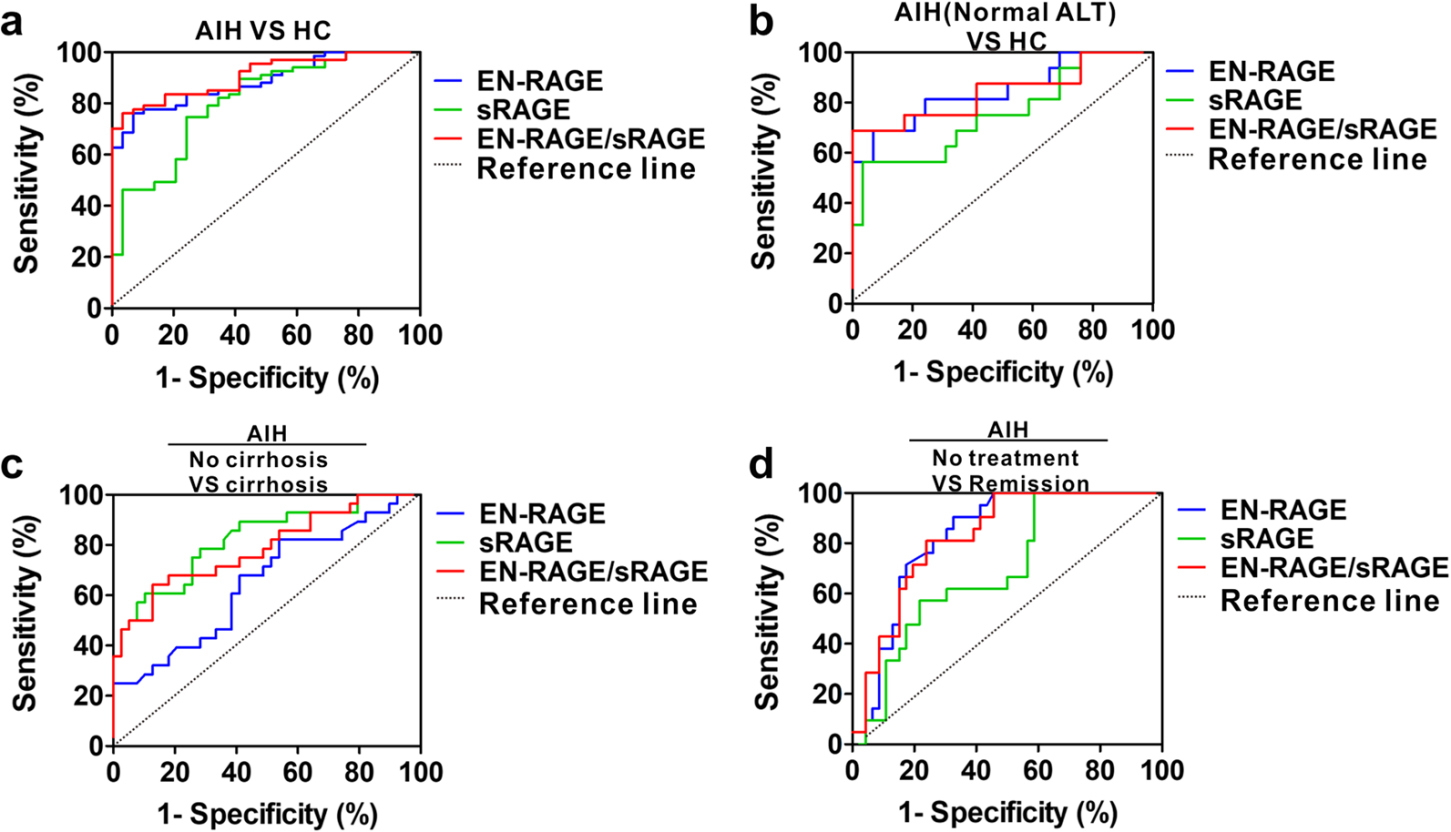
Differentiating power of serum EN-RAGE and sRAGE as well as EN-RAGE/sRAGE for AIH with different parameter. **a**, **b** ROC curves of serum EN-RAGE and sRAGE as well as EN-RAGE/sRAGE for predicting AIH **a** and AIH with nomal ALT **b** from HC. **c** ROC curves of serum EN-RAGE and sRAGE as well as EN-RAGE/sRAGE for predicting AIH with cirhosis. **d** ROC curves of serum EN-RAGE and sRAGE as well as EN-RAGE/sRAGE for predicting AIH with remission

We next evaluated differentiating power of EN-RAGE, sRAGE and EN-RAGE/sRAGE for AIH. ROC analysis revealed that EN-RAGE, sRAGE, EN-RAGE/sRAGE yielded area under the ROC curve (AUC) of 0.88 (95% CI, 0.82–0.94), 0.80 (95% CI, 0.71–0.90) and 0.90 (95% CI, 0.85–0.96), respectively (Fig. 5a), in which EN-RAGE/sRAGE with highest AUC generated 70.14% sensitivity and 96.55% specificity with cutoff value 13.08, revealing that identified EN-RAGE/sRAGE has the highest diagnostic efficacy for AIH. Hepatic biochemical necroinflammation parameter ALT does not often correlate with the liver histological findings and disease severity in AIH patients, leading to misdiagnosis and inaccurate evaluation for therapeutic effect [19]. As mentioned above that there is a correlation between EN-RAGE, sRAGE or EN-RAGE/sRAGE and ALT, we next assessed whether they have predicting power for AIH patients with normal ALT. EN-RAGE, sRAGE or EN-RAGE/sRAGE yielded AUC of 0.85 (95% CI, 0.72–0.97), 0.75 (95% CI, 0.59–0.91) and 0.83 (95% CI, 0.70–0.98), respectively (Fig. 5b), in which serum EN-RAGE with highest AUC generated 68.75% sensitivity and 93.10% specificity with cutoff value 18.73 ng/ml, indicating that identified EN-RAGE has potential predicting power for AIH patients with normal ALT. To further evaluate whether EN-RAGE, sRAGE or EN-RAGE/sRAGE can predict AIH patients with cirrhosis, we analyzed the predictive power of the three indexs in AIH patients with cirrhosis versus patients without cirrhosis. EN-RAGE, sRAGE and EN-RAGE/sRAGE yielded AUC of 0.65 (95% CI, 0.51–0.78), 0.82 (95% CI, 0.72–0.92) and 0.78 (95% CI, 0.67–0.90) (Fig. 5c), in which serum sRAGE with highest AUC generated 89.74% sensitivity and 60.71% specificity with cutoff value 254.61 pg/ml, suggesting that sRAGE could efficiently discriminate AIH patients with cirrhosis. Furthermore, EN-RAGE, sRAGE and EN-RAGE/sRAGE yielded AUC of 0.82 (95% CI, 0.75–0.92), 0.69 (95% CI, 0.56–0.82) and 0.83 (95% CI, 0.73–0.93) for identifying AIH patients with therapeutic response versus treatment-naïve AIH patients (Fig. 5d), and EN-RAGE/sRAGE with highest AUC generated 76.1% sensitivity, 81% specificity with cutoff value 36.65, suggesting that identified EN-RAGE/sRAGE also has potential predicting power for the remission of AIH patients.

## Discussion

AIH is an inflammatory liver disease characterized by liver immunotolerance failure leading to the destruction of hepatic parenchyma attacked by autoreactive T lymphocyte [14]. Elucidation of host inflammatory-immune response involved in AIH pathogenesis provides new perspectives for identification of biomarkers and therapeutic targets. EN-RAGE and sRAGE have recently gained interest because they function as important modulators in inflammatory immune response by acting on RAGE-mediated downstream cascades [5, 20]. Here, we assessed serum levels of EN-RAGE and sRAGE as well as their ratio EN-RAGE/sRAGE in AIH patients and analyzed their potential predicting power for disease progression and therapeutic response.

Growing evidence revealed that EN-RAGE/RAGE signaling pathway-mediated inflammatory-immune response play an important role in infection, autoimmunity and cancer [10, 21, 22]. However, the role of EN-RAGE/RAGE signaling in AIH-associated inflammatory-immune response has not been well-understood. Our present study demonstrated AIH patients exhibited elevated EN-RAGE and reduced sRAGE levels along with increased RAGE/sRAGE value in a well-defined cohort, indicating that EN-RAGE and sRAGE may exert opposite functions on regulating the AIH pathogenesis. This finding supports previous studies showing high EN-RAGE or low sRAGE levels in other inflammatory-immune disorders [23–25]. Based on data from assessment of differentiating power of EN-RAGE, sRAGE and EN-RAGE/sRAGE for AIH by ROC analysis, we identified EN-RAGE/sRAGE had the highest diagnostic efficacy.

The necroinflammation markers such as AST and ALT have been widely used for AIH diagnosis, prediction and monitoring disease activity [4]. Here, we observed significant correlations of EN-RAGE, sRAGE or EN-RAGE/sRAGE with these necroinflammatory indexs, implying that EN-RAGE/RAGE system activation may be involved in AIH-associated liver necroinflammatory. However, AIH patients may display normal ALT level leading to the inaccurate assessment of disease severity or even misdiagnosis [19]. In this study, we found that EN-RAGE has a predicting power for AIH patients with normal ALT. Mechanistically, AIH-associated liver injury is caused by autoimmune response to liver autoantigens, which involves a variety of immune cells, cytokines, autoantibodies and complement-mediated cytotoxicity. Various cytokines have been reported to participate in the pathogenesis of AIH. Th1 cytokine induce macrophage and T-cell activation, Th2 cytokine induce the production of autoantibodies and Th17 cytokines produce pro-inflammatory cytokines, all of which ultimately contribute to hepatocyte necroinflammation [1]. One previous in vivo study demonstrated that RAGE activation was responsible for the Th1 cell activation and cytokine production in pathogenesis of autoimmune response [15]. Secreted EN-RAGE was also reported to induce inflammatory-immune response-mediated proliferation and activation of mononuclear macrophage and lymphocyte by binding to RAGE [26]. Combining our present findings and previous studies, inflammatory-immune response triggered by EN-RAGE/RAGE ligation may participate in AIH-associated necroinflammatory activity.

Asymptomatic autoimmune hepatitis can lead to cirrhosis without treatment. Clinically, around 30% of AIH patients showed histological evidence of cirrhosis [27], suggesting early diagnosis of AIH accompanied by cirrhosis is pivotal. We found high EN-RAGE but low sRAGE levels alone with high EN/RAGE/sRAGE value in AIH with cirrhosis. Simultaneously, our present data identified that sRAGE could efficiently discriminate AIH patients with cirrhosis from without cirrhosis, generating 89.74% sensitivity and 60.71% specificity. Mechanistically, persistent AIH-mediated necroinflammation induces activation and differentiation of quiescent hepatic stellate cell and portal fibroblast into myofibroblast, a major contributor for collagen synthesis and extracellular matrix proteins (ECM) deposition, leading to the fibrous scar and terminal cirrhosis [28, 29]. Recently, elevated EN-RAGE and sRAGE were reported to involve diabetes-related cystic fibrosis and lead to worsening lung function [30]. EN-RAGE was also proven to activate fibroblast and cause dermal fibrosis by binding RAGE [31]. Therefore, we speculate that EN-RAGE/RAGE system activation might also involve in AIH-associated cirrhosis by acting on hepatic stellate cells or portal fibroblasts.

Prednisolone alone or in combination with azathioprine is the standard treatment for autoimmune hepatitis, and this treatment improves clinical, biochemical and histological features and prolongs survival in AIH patients [32]. In our present study, AIH posttreatment showed substantial normalization of clinical parameters ALT, AST and IgG. Previous study demonstrated that EN-RAGE was associated with response to therapy in juvenile idiopathic arthritis [33]. Here, we also observed an association of EN-RAGE, sRAGE or EN-RAGE/sRAGE with therapeutic effects. And a better predicting power from EN-RAGE/sRAGE was generated with AUC of 0.83, 76.1% sensitivity, 81% specificity for AIH patients with remission after prednisolone plus azathioprine treatment, suggesting that EN-RAGE/sRAGE may be potential candidate for the evaluation of therapeutic response in AIH patients.

Nonetheless, several limitations are existed in this study. Firstly, we did not assess the specificity of serum EN-RAGE and sRAGE for AIH compared to other liver autoimmune disease entities such as primary biliary cirrhosis, primary sclerosing cholangitis or their overlap syndrome. Secondly, our study was conducted in Chinese patients who were type I AIH. Further studies are required to confirm these results in type II AIH patients which mainly distributed in Europe and the United States. Finally, the detailed molecular mechanism regarding EN-RAGE/sRAGE signaling activation in AIH pathogenesis and whether sRAGE can serve as a therapeutic target still need further clarification.

## Conclusions

In conclusion, this study demonstrated that AIH patients rendered distinct patterns of serum EN-RAGE, sRAGE or EN-RAGE/sRAGE compared to healthy controls. In addition, three parameters were correlated with necroinflammation, cirrhosis incidence and therapeutic response. Furthermore, we identified EN-RAGE/sRAGE, EN-RAGE and sRAGE, had predicting power for AIH patients, AIH patients with normal ALT and cirrhosis incidence, respectively. More importantly, EN-RAGE/sRAGE also exerted predicting power for therapeutic response in AIH patients. Taken together, our findings indicate that serum EN-RAGE, sRAGE or EN-RAGE/sRAGE exhibited potentials as novel biomarkers for the diagnosis and prognosis evaluation in AIH patients, opening a very attractive field of research on these molecules in the pathology of AIH and identification of therapeutic targets.

## Supplementary information

**Additional file 1:** **Figure S1.** Serum levels of EN-RAGE and sRAGE as well as EN-RAGE/sRAGE in AIH patients with different genders.

## Data Availability

Not applicable.

## Abbreviations

AIH: Autoimmune hepatitis
HC: Healthy control
ANA: Against nuclear antigens
ASMA: Smooth muscle antibodies
LKM1: Combination of liver kidney type 1
ALT: Alanine aminotransferase
AST: Aspartate aminotransferase
IgG: Immunoglobulin G
RAGE: Receptor for advanced glycation end products
DAMP: Damage-associated molecular patterns
AGEs: Advanced glycation end products
EN-RAGE: Extracellular newly identified receptor for advanced glycation end products binding protein
sRAGE: Soluble RAGE
Th1: T helper type 1
PBC: Primary biliary cholangitis
PSC: Primary sclerosing cholangitis
ALT: Alanine aminotransferase
AST: Aspartate aminstransferase
ALB: Albumin
TB: Total bilirubin
