# Supplementary material for: Assessment of EN-RAGE, sRAGE and EN-RAGE/sRAGE as potential biomarkers in patients with autoimmune hepatitis
Source: J Transl Med. 2020 Oct 9;18:384. doi: 10.1186/s12967-020-02556-w (PMC7547460; doi:10.1186/s12967-020-02556-w)
Supplement: Supplementary file 1 — Additional file 1: Figure S1. Serum levels of EN-RAGE and sRAGE as well as EN-RAGE/sRAGE in AIH patients with different genders. [file 12967_2020_2556_MOESM1_ESM.docx]

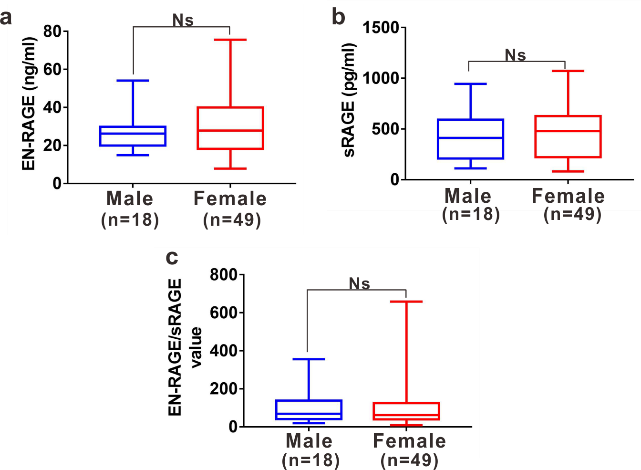


Fig.1S **Serum levels of EN-RAGE and sRAGE as well as EN-RAGE/sRAGE in AIH patients with different genders**. a Distribution of serum levels of EN-RAGE in AIH patients with different genders. b Distribution of serum levels of sRAGE in AIH patients with different genders. c Distribution of EN-RAGE/sRAGE in AIH patients with different genders. n, number. Data represents the median (IQR). Ns, no statistical significance.
